# FINANCIAL HORIZONS: EXAMINING RURAL AND URBAN NURSING HOME FINANCIAL PERFORMANCE

**DOI:** 10.1093/geroni/igae098.0418

**Published:** 2024-12-31

**Authors:** Gregory Orewa, Akbar Ghiasi, Shivani Gupta, Rohit Pradhan, Robert Weech-Maldonado

**Affiliations:** The University of Texas San Antonio, San Antonio, Texas, United States; University of the Incarnate Word, San Antonio, Texas, United States; University of Houston - Clear Lake, Houston, Texas, United States; Texas State University, San Marcos, Texas, United States; University of Alabama at Birmingham, Birmingham, Alabama, United States

## Abstract

Rural nursing homes (NH) struggle with financial sustainability due to sparse populations, high costs, and scarce healthcare resources. Urban counterparts face increased operational costs and resource competition, despite larger populations and better infrastructure. Addressing these distinct challenges is essential for the sector’s financial health and strategic relevance. This study examines financial performance differences between rural and urban nursing homes. The study utilized secondary data from 2017 to 2022 from the Medicare Cost Report, LTC Focus, Care Compare, and Area Health Resource File. The data were modeled using hierarchical linear regression controlling for year and state fixed effects. The dependent variable was operating margin, and the independent variable was nursing home location (Rural vs. Urban). Two models were run, the first controlling for organizational factors (size, quality, ownership, occupancy rate, payer mix, and percent minorities) and the second for organizational and environmental factors (population 65 and older, competition, and Medicare Advantage) that may be associated with financial performance. Results suggest that urban nursing homes had significantly higher operating margins compared to rural facilities (β=1.95, p< 0.01) after controlling for organizational factors. After controlling for environmental factors, urban nursing homes still had higher operating margins (β=0.90, p< 0.01) than rural facilities, but to a lesser extent. As such, rural nursing homes have lower financial performance than urban, even after accounting for organizational and environmental differences. To the extent that lower financial performance may affect quality and access to long-term care, policymakers should explore reimbursement strategies to address these financial disparities.

